# Novel Target for Pathogenic Leptospira Detection via Colorimetric Loop‐Mediated Isothermal Amplification (LAMP): Development, Validation, and Comparative Analysis

**DOI:** 10.1002/jcla.70277

**Published:** 2026-10-14

**Authors:** Peyman Ghoraishizadeh, Benjie Clemente, Kween Saimuang, Sakaorat Lertjuthaporn, Sukhonta limsampan, Patcharapan Suwannin, Mongkol Techakasikornpanich, Galayanee Doungchawee, Edilberto Manahan, Kulachart Jangpatarapongsa

**Affiliations:** ^1^ Center for Research Innovation and Biomedical Informatics, Faculty of Medical Technology Mahidol University Nakhon Pathom Thailand; ^2^ Department of Medical Technology, Faculty of Pharmacy University of Santo Tomas Manila Philippines; ^3^ The Graduate School, University of Santo Tomas Manila Philippines; ^4^ School of Health Sciences University of Santo Tomas General Santo Philippines

**Keywords:** LAMP, leptospirosis, novel target, pathogenic *Leptospira*, real‐time PCR

## Abstract

**Background:**

Leptospirosis is a globally prevalent zoonotic disease that remains challenging to diagnose due to its nonspecific symptoms and the limitations of current diagnostic methods and targets. To address these challenges, we developed and validated a naked‐eye loop‐mediated isothermal amplification (LAMP) assay targeting the newly identified orpH region for the rapid detection of pathogenic Leptospira species.

**Methods:**

Comparative genomic analysis identified orpH as a conserved sequence among pathogenic Leptospira. A colorimetric LAMP assay (LAMP‐orpH) was developed and evaluated using reference strains and serum samples obtained from patients with suspected leptospirosis. The diagnostic performance of the assay on these serum samples was evaluated in comparison with conventional PCR and the microscopic agglutination test (MAT), with the real‐time PCR assay targeting lipL32 designated as the reference standard.

**Results:**

The LAMP‐*orpH* assay demonstrated 87.2% sensitivity and 94.8% specificity among 76 suspected cases and 29 controls, showing strong agreement with real‐time PCR (*κ* = 0.826). It outperformed MAT (63.8% sensitivity) and conventional PCR (23.4% sensitivity), achieving a detection limit of 10^2^ cells/mL in spiked serum.

**Conclusion:**

The LAMP‐*orpH* assay offers a rapid, sensitive, and specific tool for early leptospirosis diagnosis. Its simple colorimetric readout and minimal equipment requirements make it ideal for use in rural and resource‐limited settings, providing a practical alternative to MAT, conventional PCR, and qPCR.

## Graphical abstract

A novel colorimetric LAMP assay targeting the org‐hp gene was developed for rapid Leptospira detection. The assay is performed in a simple water bath at 65°C for 90 min, and results can be interpreted by a color change visible to the naked eye. This method provides a sensitive, specific, and field‐adaptable diagnostic tool for use in resource‐limited settings.

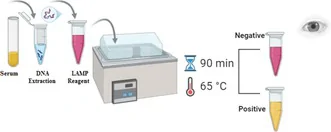

## 1 Introduction

Leptospirosis is one of the most prevalent zoonotic diseases, significantly affecting both humans and livestock [1]. The disease is caused by *Leptospira* spp., which were traditionally categorized into three clades: pathogenic, saprophytic, and intermediate, each exhibiting varying levels of pathogenicity. Recent genomic studies have refined this classification into two major clades (Pathogens P and Saprophytes S) and four subclades: P1 (pathogens), P2 (intermediates), S1 (saprophytes), and S2 (a newly described saprophytic‐related group), providing a more accurate taxonomy that avoids assumptions about virulence. The P1 subclade causes a broad spectrum of human illnesses, ranging from asymptomatic infection to severe conditions with potentially rapid mortality [2, 3, 4].

Globally, leptospirosis affects 10 to 100 cases per 100,000 annually with more than 1.03 million cases and approximately 58,900 deaths reported each year [5, 6]. The disease poses heightened risks in tropical and subtropical regions, where environmental and occupational exposure to *Leptospira* spp. is common. Southeast Asia, with its humid climate and extensive agricultural activity, has the second‐highest global incidence of leptospirosis, emphasizing the urgent need for improved diagnostic tools in endemic areas [7]. The clinical manifestation of leptospirosis is nonspecific, often mimicking other febrile diseases prevalent in the same geographic regions, delaying diagnosis and appropriate treatment. While leptospirosis is curable with prompt antibiotic therapy, the absence of rapid and reliable diagnostic assays remains a major barrier to timely disease management. Traditional diagnostic methods, including culture and the Microscopic Agglutination Test (MAT), are considered gold standards but are time‐consuming and poorly suited for early diagnosis [6]. Culture typically requires several weeks for bacterial growth, while MAT relies on the host's antibody response, thereby reducing its sensitivity during the acute phase of infection [8]. Molecular diagnostic techniques, such as polymerase chain reaction (PCR) and quantitative real‐time PCR (qPCR), can detect *Leptospira*‐specific DNA with high accuracy but are constrained by cost, equipment requirements, and the need for skilled personnel, making them impractical for many laboratories in resource‐limited settings [9, 10, 11]. These limitations highlight the urgent need for the development of a rapid, cost‐effective, and reliable point‐of‐care diagnostic assay for leptospirosis to facilitate early detection and improve clinical outcomes in endemic regions.

Loop‐mediated isothermal amplification (LAMP), first introduced by Notomi et al. in 2000, is a rapid, simple, and cost‐effective nucleic acid amplification technique that amplifies DNA under constant temperature conditions (60°C–65°C). LAMP provides high analytical sensitivity and specificity without the need for thermocycling, making it particularly suitable for point‐of‐care diagnostics and laboratories with limited infrastructure [12, 13]. Despite its advantages, relatively few studies have optimized or validated LAMP assays for leptospirosis detection in endemic regions [14, 15, 16].

Selecting appropriate genetic targets and designing robust primers are critical for the success of molecular diagnostic assays, as they determine assay specificity and sensitivity in detecting microorganisms, particularly in clinical samples [13, 17]. Accurate detection of pathogenic *Leptospira* depends critically on the selection of an appropriate genetic target. Numerous genes have been evaluated for diagnostic applications, each with distinct limitations [18, 19, 20], for instance, the *secY* gene is conserved across all *Leptospira* species and therefore cannot reliably differentiate pathogenic from saprophytic clades [14]. Similarly, the *rrs* gene exhibits high sensitivity but amplifies DNA from both P1 and P2 subclades, reducing its diagnostic specificity [15]. Although the *lipL32* gene is regarded as highly specific to the P1 subclade and frequently employed in commercial diagnostic kits, its reported sensitivity has varied considerably among studies [10, 16, 21]. This study aimed to identify and validate a novel genetic target for the detection of pathogenic Leptospira species. Through comparative genomic analysis, a highly conserved sequence designated orpH was identified as a promising diagnostic target. A phenol red‐based colorimetric loop‐mediated isothermal amplification (LAMP) assay was subsequently developed to enable rapid, simple, and equipment‐free visual detection of Leptospira DNA. The optimized LAMP assay targeting the orpH sequence (LAMP‐orpH) was evaluated alongside conventional PCR targeting the same sequence (PCR‐orpH) and the microscopic agglutination test (MAT), with a commercial real‐time PCR assay targeting lipL32 serving as the reference standard. Human serum samples from suspected cases of leptospirosis were analyzed to determine the diagnostic sensitivity, specificity, and accuracy of each method, providing a comprehensive comparative assessment of their performance in detecting pathogenic Leptospira.

## 2 Materials and Methods

### 2.1 Bioinformatics Study and PCR Primer Designing

A modified comparative genomic analysis was conducted to identify a novel DNA target specific to pathogenic Leptospira [22]. Briefly, the complete genome sequence of *Leptospira interrogans* strain NEG7 (NZ_CP093938.1) was selected as the reference. A total of 4,297,584 nucleotide base pairs were retrieved and segmented into independent fragments of 5000 bp each. Each fragment was then individually analyzed using the Basic Local Alignment Search Tool (BLAST) to compare with other nucleotide sequences available in the collection database including complete genome and whole‐genome shotgun sequences. Candidate fragments were shortlisted based on sequence specificity to pathogenic Leptospira. Filtration was performed according to two criteria: (i) fragments exhibiting ≥ 90% query coverage and sequence identity with Leptospira species, and (ii) fragments showing ≤ 50% query coverage with any non‐Leptospira microorganism.

Final alignment and validation of conserved regions were performed using ClustalW in BioEdit version 7.0.5.3 (Ibis Biosciences, Carlsbad, CA, USA) (Figure S1). Sequences exhibiting ≥ 99% identity with pathogenic Leptospira and no significant similarity with saprophytic or intermediate species were defined as Leptospira‐conserved sequences. The selected conserved region, designated orpH, was subsequently used for primer design for both PCR‐orpH and LAMP‐orpH assay development.

### 2.2 DNA Isolation and PCR Optimization

Pathogenic *Leptospira* species were cultured in liquid Ellinghausen and McCullough medium, modified by Johnson and Harris (EMJH), following standardized protocols [23, 24], *Leptospira interrogans* serovar Canicola was selected from our laboratory's *Leptospira* strain bank because it is a representative pathogenic P1 strain commonly found in Southeast Asia. The identity of the isolate was verified by the Microscopic Agglutination Test (MAT) using serovar‐specific antisera prior to genomic DNA extraction. Genomic DNA was extracted using the Presto Mini gDNA Bacteria Kit (Geneaid Biotech Ltd., Thailand) according to the manufacturer's instructions. DNA concentrations were measured using a NanoDrop One/Onec Microvolume UV–Vis Spectrophotometer (Thermo Scientific, USA), and the purified DNA was stored at −20°C until further use.

For PCR‐orpH assays, 1 ng of extracted DNA and nuclease‐free water were used as positive and negative controls, respectively. The 25 μL reaction mixture consisted of 12.5 μL AccuStart II GelTrack PCR SuperMix (containing 2.5 mM MgCl_2_, 200 μM dNTPs, and 0.2 U/μL DNA polymerase) (Quantabio, Germany), 5.5 μL sterile water, 1 μL of each forward and reverse primer (Table 2), and 5 μL of DNA template. Amplification of PCR‐orpH was performed under the following thermal cycling conditions: an initial denaturation step at 94°C for 5 min, followed by 40 cycles of denaturation at 94°C for 45 s, annealing at 59°C for 30 s, and extension at 72°C for 30 s; and a final extension step at 72°C for 10 min. PCR products were analyzed by electrophoresis on a 2% agarose gel prepared with Tris‐acetate‐EDTA (TAE) buffer visualized with the Gel Doc EZ System (Biorad, USA).

### 2.3 Analytical Specificity and Sensitivity of PCR Primers

The analytical specificity of the designed primers was initially assessed in silico using the *blastn* against all available databases to evaluate any cross‐reactivity with bacterial or human genomes. Experimental validation was subsequently performed using a panel of 19 pathogenic *Leptospira* strains P1, one intermediate *Leptospira* species *(L. fainei, P2)* purchased from Amsterdam University Medical Center (Netherlands), one saprophytic *Leptospira strain* ( *L. biflexa* , S1), and five additional microorganisms, as detailed in Table 1. These non‐Leptospira microorganisms were selected for their diagnostic relevance, as they cause infections with clinical manifestations like leptospirosis such as typhus, malaria, and meningitis. The intermediate species was included due to its potential to cause misidentification in routine diagnostic testing [25, 26]. *Treponema pallidum* , a species within the same order (*Spirochaetales*) as *Leptospira*, was also included to assess potential cross‐reactivity and confirm the assay's specificity for *Leptospira* detection.

**TABLE 1:** List of bacteria used in this study.

No	Species	Serovars	Strains	Clade	No	Species	Serovars	Strains/genome	Clade
1	*L. interrogans*	Canicola	ATCC	P1	14	*L. borgpetersenii*	Hardjo	L550	P1
2	*L. interrogans*	Hebdomadis	Hebdomadis	P1	15	*L. borgpetersenii*	Tarassovi	Perepelitsin	P1
3	*L. interrogans*	Bataviae	Swart	P1	16	*L. kirschneri*	Cynopteri	3522 C	P1
4	*L. interrogans*	Djasiman	Djasiman	P1	17	*L. borgpetersenii*	Ballum	Mus 127	P1
5	*L. interrogans*	Bratislava	Jez Bratislava	P1	18	*L. borgpetersenii*	Javanica	Veldrat Batavia 46	P1
6	*L. santarosai*	Shermani	1342 K	P1	19	*L. kirschneri*	Grippotyphosa	ATCC	P1
7	*L. noguchii*	Ranarum	ICF	P1	20	*L. biflexa*	Patoc	NAIH	S1
8	*L. noguchii*	Louisiana	LSU 1945	P1	21	*L. fainei*	Hurstbridge	BUT 6	P2
9	*L. noguchii*	Panama	MBC	P1	22	*Escherichia coli*		ETEC	
10	*L. interrogans*	Copenhageni	M20	P1	23	*Pseudomonas aeruginosa*		B420	
11	*L. interrogans*	Autumnalis	Akiyami A	P1	24	*Salmonella typhi*		Ty2	
12	*L. interrogans*	Pomona	Pomona	P1	25	*Plasmodium vivax*		PvW1	
13	*L. interrogans*	Australis	Ballico	P1	26	*Treponema pallidum*		Nicholas	

Positive PCR products were selected for DNA sequencing using a commercial service (ATGC Co. Ltd., Thailand). The resulting sequences were analyzed using Chromas Version 2.5, and BLAST analysis was performed to identify homology with sequences in the NCBI database.

To evaluate the analytical sensitivity of the primers, PCR was performed on serial dilutions of the *L. interrogans* serovar Canicola genomic DNA prepared in nuclease‐free water (Thermo Fisher Scientific, USA) ranging from 100 ng/μL to 10 fg/μL.

The *lipL32* gene, a well‐established target commonly used in commercial kits for pathogenic *Leptospira* detection, served as a reference. The analytical specificity and sensitivity of the primers designed for the new target PCR‐orpH were compared with those of the *lipL32* primers PCR‐lipL32, which produce an expected amplicon size of 474 bp, as previously described [27].

### 2.4 Development of a Colorimetric LAMP Assay

A colorimetric loop‐mediated isothermal amplification (LAMP) assay targeting the *orpH* sequence was developed using four primers designed with the PrimerExplorer V5 online software (https://primerexplorer.jp/e/). The reactions were performed using theWarmStart Colorimetric LAMP 2× Master Mix (DNA & RNA) (New England Biolabs; MA, USA) contains phenol red as a pH indicator enabling naked‐eye detection through a visible color shift from pink to yellow Each 25 μL reaction consisted of 2 μL of genomic DNA (concentration range: 5–50 ng/μL), 12.5 μL of WarmStart Colorimetric LAMP Master Mix, 0.5 μL of 10 μM each of outer primers (F3 and B3), 1 μL of 40 μM each of inner primers (FIP and BIP) and nuclease‐free water to reach the final volume. The reactions were incubated in a water bath (Esstell, Estell Co Ltd., Korea) at a constant isothermal temperature of 60°C–65°C for 30–90 min to determine optimal amplification conditions. The analytical sensitivity of LAMP assay was determined using tenfold serial dilutions of *L. interrogans* serovar Canicola genomic DNA (100 ng/μL to 1 fg/μL). Detection was assessed based on the colorimetric change and confirmed by agarose gel electrophoresis. Analytical Specificity testing was performed using the bacterial panel described in Table 1.

To evaluate the limit of detection (LOD) of the LAMP‐orpH assay in a clinical sample model, *Leptospira interrogans* serovar Canicola was used for spiking experiments. The concentration of leptospiral cells was accurately determined using a Petroff–Hausser counting chamber under dark‐field microscopy. Subsequently, serial tenfold dilutions ranging from 1 × 10^6^ to 1 cell/mL were prepared in human serum. DNA was extracted from 200 μL of each spiked serum sample and eluted in 50 μL buffer using the QIAamp DNA Blood Mini Kit (QIAGEN, Germany) and analyzed by the LAMP assay to assess limit of detection.

### 2.5 Microscopic Agglutination Test (MAT)

The Microscopic Agglutination Test (MAT) was performed according to the standardized protocol described previously [28, 29] In brief, serial twofold dilutions of patient sera were incubated with live *Leptospira* reference antigens representing major pathogenic serogroups and examined under a dark‐field microscope for agglutination. A reciprocal antibody titer of ≥ 1:800 was considered indicative of a positive reaction for leptospiral infection.

### 2.6 Clinical Study

From April to December 2024, a cohort of 302 consecutive patients presenting with febrile illness was evaluated at Ilocos Training and Regional Medical Center and Quezon City General Hospital. Among them, 76 individuals met the inclusion criteria, which required a documented history of fever or a temperature of ≥ 38°C, accompanied by at least one additional symptom, such as headache, rash, myalgia, arthralgia, lymphadenopathy, meningitis, encephalitis, respiratory symptoms, jaundice, or acute renal failure. Exclusion criteria included patients below 6 months of age, fever lasting beyond 1 month, or an admission diagnosis of wound infection, diabetic foot infection, postoperative infection, abscess, parotitis, urinary infection, or diarrhea.

Anonymized samples were utilized in this study, which received ethical approval from the Region I Medical Center, the Institutional Review Board—REC Philippines (reference number: 2024‐027). DNA was extracted from serum samples of 76 patients and 29 healthy controls using the QIAGEN DNA extraction kit. The serum was employed for the MAT assay, while the extracted DNA underwent analysis using both LAMP and PCR assays. The results obtained from these assays were then compared with those derived from the BactoReal Kit Leptospira spp. (lipL3232) (Ingenetix), a commercially available real‐time PCR assay specifically designed for the detection of pathogenic *Leptospira* by targeting the lipL3232 gene.

Specificity, sensitivity, positive predictive value (PPV), negative predictive value (NPV), and accuracy were calculated along with 95% Clopper‐Pearson confidence intervals (CI). The agreement between the colorimetric LAMP‐orpH, MAT, and PCR‐orpH assays was evaluated against the qPCR kit, considered the reference standard, using Cohen's kappa coefficient to quantify the level of concordance between the methods. Data analysis was performed using IBM SPSS Statistics version 27.

## 3 Results

### 3.1 Bioinformatics Study and PCR Primer Design

The orpH sequence, comprising an oxidoreductase gene segment and the terminal region of a hypothetical protein, was identified through comparative genomic analysis (Figure S2) as a unique and highly conserved region specific to pathogenic Leptospira species. This sequence was selected as a novel molecular target for diagnostic assay development. Primers targeting the orpH region were designed for both PCR‐orpH and LAMP‐orpH assays, optimized to minimize secondary structure formation and ensure high amplification efficiency. The nucleotide sequences of all primers used in this study are listed in Table 2, and the expected amplicon size for the PCR‐orpH assay was 290 base pairs.

**TABLE 2:** The list of primers used in this study.

Number	Primer name	Oligo sequences 5′ → 3′	References
1	PCR‐orpH F	CTGGTTTGAGAGTAAAAGCGGA	This study
2	PCR‐OrpH R	CCTACACTGCGTGACCGTAAG	This study
3	PCR lipL32 F	ATCTCCGTTGCACTCTTTGC	[20]
4	PCR lipL32‐R	ACCATCATCATCATCGTCCA	[20]
5	LAMP‐orpH F3	GGCTTCAAAACTCTTTAGGT	This study
6	LAMP‐orpH B3	AAATGCACCAAGAAAAAATGG	This study
7	LAMP‐orpH FIP	GCCTATGGGTGAAATTCTTCCTTAT‐TTTT‐AGATTTTTCACCATGTAGATTTCG	This study
8	LAMP‐orpH BIP	AATTCACAGTTTACTTGCATACGGC‐TTTT‐TCCAAAAGATCCGGAGAAG	This study

### 3.2 The Analytical Specificity and Sensitivity Testing of PCR

The analytical specificity of the *orpH* primers was evaluated using DNA extracted from 19 pathogenic *leptospira* serovars (P1), one saprophytic (S1) and intermediate Leptospira (P2), and 5 other non‐Leptospira microorganisms demonstrated exclusive amplification of pathogenic leptospira DNA. No amplification was observed with DNA from saprophytic and intermediate *leptospira* or other non‐leptospira microorganisms. Gel electrophoresis of PCR products confirmed 100% specificity for both PCR‐orpH (290 bp) and PCR‐lipL32 (474 bp) primers (Figure S3). Sequencing of representative PCR amplicons showed complete alignment with pathogenic *Leptospira* reference sequences available in the GenBank database.

Analytical sensitivity analysis, performed using serial tenfold dilutions of *leptospira interrogans* serovar Canicola genomic DNA (ranging from 100 ng/μL to 10 fg/μL), showed that the PCR‐*orpH*assay achieved a detection limit of 1 pg/μL, demonstrating 10 fold higher sensitivity than PCR‐lipL32 assay, which had an LOD of 10 pg/μL in triplicate experiments (Figure S3).

### 3.3 Optimization of LAMP Condition

In this study, four parameters influencing the LAMP reaction were evaluated to optimize the detection of pathogenic *leptospira*. These parameters included the concentrations of external primers (0.05–0.4 μM) and internal primers (1.2–2.0 μM), as well as reaction temperature (60°C–65°C) and incubation time (30–90 min). Optimal amplification was achieved using 1.6 μM internal primers and 0.2 μM external primers at 65°C for 90 min. Under these conditions, the colorimetric LAMP assay produced the most distinct and consistent color change from pink to yellow, indicating robust amplification (Figures 1 and 2). The LAMP‐orpH assay achieved a detection limit of 100 fg/μL, as confirmed by both colorimetric analysis and gel electrophoresis (Figure S4). No amplification was observed at 10 fg/μL or 1 fg/μL, defining the lower detection threshold. The LAMP‐orpH showed high analytical specificity, producing amplification only with pathogenic *Leptospira* DNA and none with saprophytic or non‐*Leptospira* species (Figure S5). In spiked serum samples, the LAMP‐*orpH* assay detected as few as 10^2^ *Leptospira* cells/mL, confirming its sensitivity and suitability for complex biological matrices. All experiments were performed in triplicate.

**FIGURE 1:**
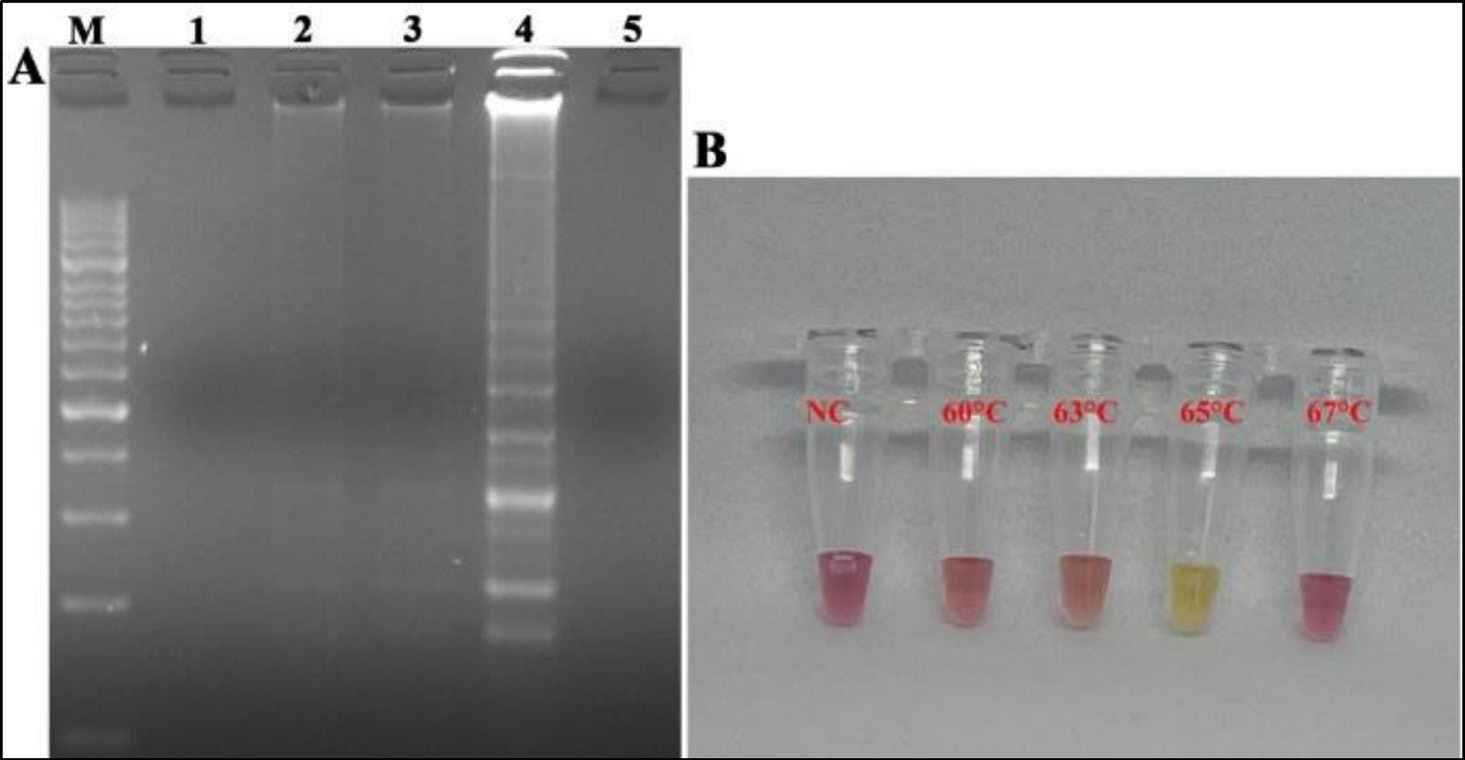
Optimized conditions for the colorimetric LAMP assay at various temperatures. (A) Gel electrophoresis results of LAMP amplification. M: 100 bp DNA marker, lane 1: No‐template control, lane 2: 60°C, lane 3: 63°C, lane 4: 65°C, lane 5: 67°C. (B) Visual color change from pink to yellow at various temperatures where NC was no‐ template control.

**FIGURE 2:**
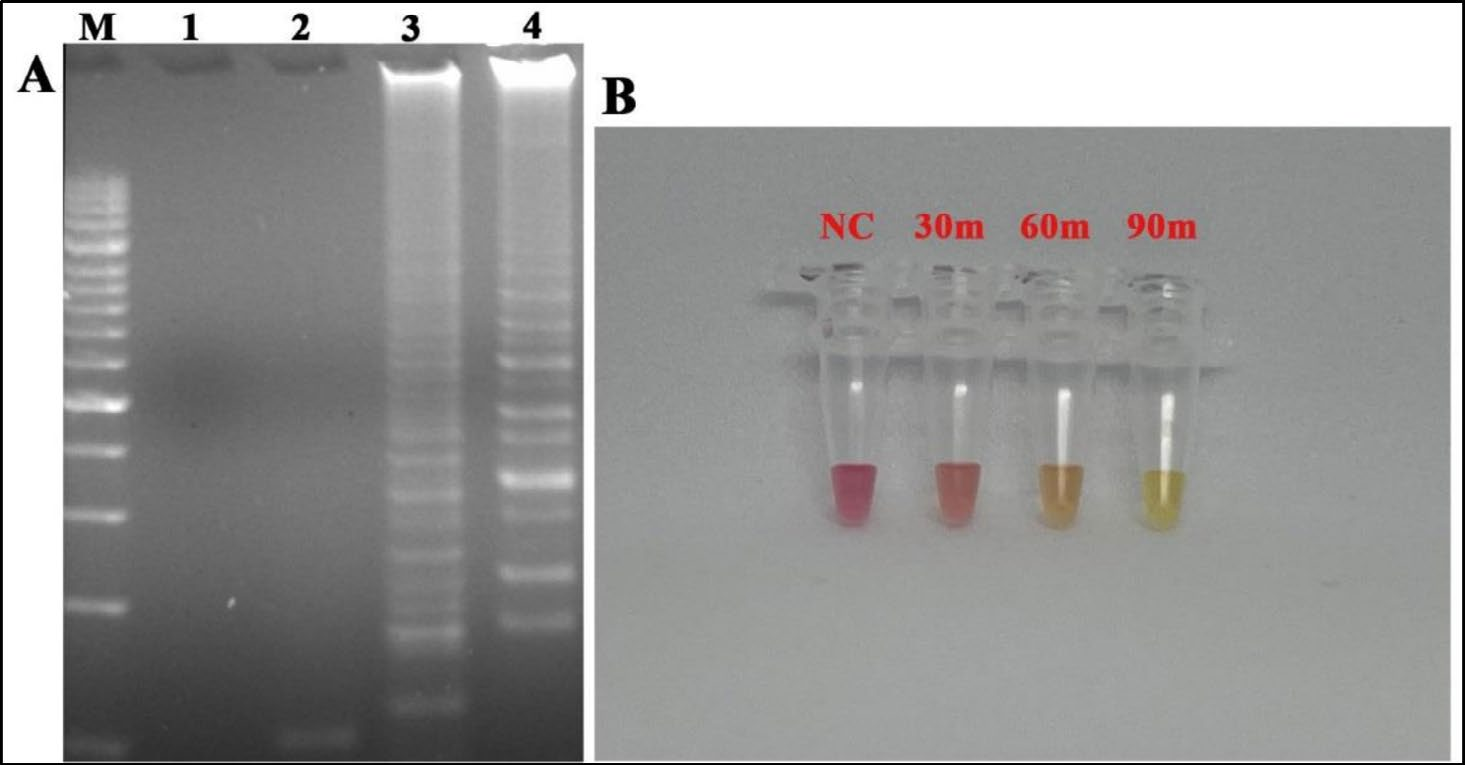
Optimized conditions for the colorimetric LAMP assay at various times. (A) Gel electrophoresis results of LAMP amplification. M: 100 bp DNA Marker, lane 1: No‐template control, lane 2: 30 m, lane 3: 60 m, lane 4: 90 m. (B) Visual color change from pink to yellow at various temperatures, where NC was the no‐template control.

### 3.4 Performance of LAMP Assay in Clinical Samples

The diagnostic performance of the LAMP‐*orpH*, *PCR‐orpH*, and MAT assays was evaluated in comparison with the *lipL32* qPCR assay, which served as the reference standard. Among the 76 clinical samples, 47 were qPCR‐positive and 29 were qPCR‐negative, with an additional 29 negative samples obtained from healthy controls.

The LAMP‐orpH assay identified 41 true‐positive samples with six false‐negative and three false positives, yielding a sensitivity of 87.23% and a specificity of 94.83%. The *PCR‐orpH* assay detected 12 positive samples, including one false positive and 36 false negatives, corresponding to a sensitivity of 23.40% and a specificity of 98.28%. The microscopic agglutination test (MAT) detected 31 positive samples, with one false positive and 17 false negatives, demonstrating a sensitivity of 63.83% and a specificity of 98.28%. All individual sample results for MAT, LAMP, and conventional PCR in comparison with real‐time PCR are summarized in Table 3.

**TABLE 3:** Individual results of clinical samples tested by MAT, LAMP, and conventional PCR compared with real‐time PCR.

No	Code	Real‐time PCR	LAMP orpH	PCR orpH	MAT/titer
1	P1	+	+	−	+/1:800
2	P2	+	−	−	−
3	P3	+	+	−	+/1:800
4	P4	+	+	−	+/1:800
5	P5	+	+	+	+ /1:800
6	P6	+	+	−	+/1:800
7	P7	+	−	−	−
8	P8	+	+	−	+ /1:800
9	P9	+	−	−	−
10	P10	+	+	−	−
11	P11	+	+	+	+ /1:800
12	P12	+	+	−	+/1:800
13	P13	+	+	−	+/1:800
14	P14	+	−	−	+/1:800
15	P15	+	+	−	+/1:800
16	P16	+	+	+	−
17	P17	+	+	−	+/1:800
18	P18	+	+	−	+/1:800
19	P19	−	−	−	+/1:800
20	P20	−	−	−	−
21	QCGH008	+	+	−	+/1:800
22	QCGH 011	−	−	−	−
23	QCGH017	+	+	+	−/1:400
24	QCGH025	−	+	−	−
25	P25	−	−	−	−
26	P31	+	+	−	+/1:800
27	P050	−	−	−	−
28	P37	+	+	+	−
29	P39	−	−	−	−
30	QCGH067	−	−	−	−
31	QCGH067	Not valid	Not valid	Not valid	Not valid
32	QCGH059	−	−	−	−
33	QCGH052	+	+	+	+/1:800
34	P30	−	−	−	−
35	QCGH080	−	−	−	−
36	QCGH114	−	−	−	−
37	QCGH132	+	+	+	+/1:1600
38	QCGH142	−	−	−	−
39	QCGH151	−	−	−	−
40	QCGH155	+	+	−	+/1:800
41	QCGH156	−	−	−	−
42	QCGH163	−	−	−	−
43	QCGH167	−	−	−	−
44	QCGH174	+	+	−	−
45	QCGH189	−	−	−	−
46	QCGH162	+	+	+	−
47	QCGH028	+	+	−	+/1:800
48	ITRMC 1	+	+	−	−
49	ITRMC 2	+	−	−	−
50	ITRMC 3	−	−	−	−
51	ITRMC 4	−	−	−	−
52	ITRMC 11	+	+	+	+1/800
53	ITRMC 18	+	+	−	−
54	ITRMC 21	+	+	+	+1/800
55	ITRMC 38	+	+	−	+1/800
56	RMC 1	−	−	−	−
57	RMC 2	−	−	−	−
58	RMC3	+	−	−	−
59	RMC5	+	+	+	+1/800
60	RMC7	−	+	+	−
61	RMC8	+	+	−	+1/1600
62	RMC12	−	−	−	−
63	CVMC2	+	+	−	+1/800
64	CVMC3	+	+	−	+1/800
65	CVMC4	+	+	−	+1/1600
66	CVMC 5	+	+	−	+1/800
67	CVMC6	−	+	−	−
68	CVMC7	−	−	−	−
69	CVMC8	+	+	−	+1/800
70	CVMC9	−	−	−	−
71	CVMC11	+	+	−	−
72	CVMC12	+	+	−	−
73	CVMC14	+	+	−	−
74	CVMC15	+	+	−	−
75	CVMC16	+	+	−	+1/800
76	CVMC17	−	−	−	−
77	CVMC 19	−	−	−	−
78	N1	−	−	−	−
79	N2	−	−	−	−
80	N3	−	−	−	−
81	N4	−	−	−	−
82	N5	−	−	−	−
83	N6	−	−	−	−
84	N7	−	−	−	−
85	N8	−	−	−	−
86	N9	−	−	−	−
87	N10	−	−	−	−
88	N11	−	−	−	−
89	N12	−	−	−	−
90	N13	−	−	−	−
91	N14	−	−	−	−
92	N15	−	−	−	−
93	N16	−	−	−	−
94	N17	−	−	−	−
95	N18	−	−	−	−
96	N19	−	−	−	−
97	N20	−	−	−	−
98	N21	−	−	−	−
99	N22	−	−	−	−
100	N23	−	−	−	−
101	N24	−	−	−	−
102	N25	−	−	−	−
103	N26	−	−	−	−
104	N27	−	−	−	−
105	N28	−	−	−	−
106	N29	−	−	−	−

Agreement with the qPCR reference, measured using Cohen's kappa coefficient (*κ*), was poor for *PCR‐orpH* (*κ* = 0.233), good for MAT (*κ* = 0.642), and strong for LAMP‐*orpH* (*κ* = 0.826). A detailed summary of diagnostic outcomes is presented in Table 4.

**TABLE 4:** Performance comparison of colorimetric LAMP‐*orpH*, PCR‐*orpH*, and MAT compared with the real‐time PCR kit results.

Molecular test		Real‐time PCR		Performances	Results	95% CI
		Positive	Negative			
LAMP‐orpH	Positive	41	6	Sensitivity	87.23%	74.26%–95.17%
	Negative	3	55	Specificity	94.83%	85.62%–98.92%
				PPV	93.18%	81.87%–97.64%
				NPV	90.16%	81.24%–95.10%
				Accuracy	91.43%	84.35%–96.01%
				Kappa value^a^ (*p* < 0.001)	0.826	0.717 to 0.934
MAT	Positive	30	17	Sensitivity	63.83%	48.52%–77.33%
	Negative	1	57	Specificity	98.28%	90.76%–99.96%
				PPV	96.77%	80.94%–99.53%
				NPV	77.03%	69.60%–83.08%
				Accuracy	82.86%	74.27%–89.51%
				Kappa value^a^ (*p* < 0.001)	0.642	0.499–0.784
PCR‐orpH	Positive	11	36	Sensitivity	23.40%	12.30%–38.03%
	Negative	1	57	Specificity	98.28%	90.76%–99.96%
				PPV	91.67%	59.56% to 98.80%
				NPV	61.29%	57.39%–65.05%
				Accuracy	64.76%	54.83%–73.84%
				Kappa value^a^ (*p* < 0.001)	0.233	0.097–0.369

Abbreviations: NPV, negative predictive value; PPV, positive predictive value.

^a^ The kappa value is a measure of test reliability, with the values interpreted as follows: < 0.4 = poor; 0.4 to 0.75 = fair to good; > 0.75 = strong.

## 4 Discussion

Leptospirosis remains one of the most common causes of acute febrile illnesses and is recognized as the most prevalent zoonotic disease globally The genus *Leptospira* encompasses more than 65 species and over 300 serovars, demonstrating remarkable genetic and antigenic diversity [2, 3], This diversity poses significant challenges for accurate diagnosis and surveillance, often leading to underreporting and misclassification of cases, particularly in endemic regions. Furthermore, the clinical manifestation of leptospirosis is nonspecific and often indistinguishable from other infectious diseases prominent in the same geographic regions, such as dengue fever, malaria, and scrub typhus, frequently resulting in delayed or incorrect diagnosis [30, 31]. Early and accurate diagnosis during the leptospiremic phase is essential to enable timely administration of both specific treatments and appropriate supportive care. However, rapid confirmation of the disease remains a major clinical challenge. Early and accurate diagnosis during the leptospiremic phase is essential to enable timely administration of specific antimicrobial therapy and appropriate supportive care. However, rapid confirmation of infection remains a major clinical challenge. Direct microscopic examination of clinical specimens for *Leptospira* is limited by poor sensitivity, low specificity, and operator subjectivity. Molecular assays and serological tests, including nucleic acid amplification and antibody‐based methods, are also used for early detection but each presents inherent limitations in terms of diagnostic accuracy, turnaround time, and accessibility. Therefore, prompt and reliable identification of the causative pathogen remains critical for ensuring optimal patient management and improving clinical outcomes.

The continuous improvement and development of diagnostic tools are fundamental to microbiological practice, as they reduce the likelihood of misdiagnosis and enhance clinical decision‐making. Among molecular approaches, loop‐mediated isothermal amplification (LAMP) has gained prominence as a rapid and cost‐effective diagnostic technique, distinguished by its high specificity, sensitivity, and rapid amplification under isothermal conditions [32, 33]. Unlike conventional PCR, LAMP does not require thermocycling or sophisticated equipment, making it particularly suitable for field deployment and use in resource‐limited laboratories [34]. Over the past decade, substantial efforts have focused on adapting LAMP‐based assays for the detection of *Leptospira*, with promising results reported in both human and veterinary studies [12, 15, 35, 36]. These developments are especially relevant in tropical and subtropical regions where leptospirosis is endemic, and laboratory infrastructure is often limited. Notably, more than half of published studies on leptospirosis diagnostics approximately 54% originate from Asian countries, highlighting both the disease burden and the regional demand for reliable, rapid diagnostic platforms [18]. Despite this progress, variability in target gene selection and assay validation continues to hinder standardization across laboratories, reinforcing the need for new, robust genetic markers that can improve diagnostic accuracy and global applicability.

The selection of an appropriate genetic target is fundamental to improving the sensitivity and specificity of molecular diagnostic assays. However, currently available targets for detecting pathogenic *Leptospira* remain limited. For example, the *secY* gene, although frequently employed in leptospiral detection, is present across all *Leptospira* species and lacks discrimination between pathogenic and non‐pathogenic strains. Indeed, amplification of *secY* has been reported in the saprophytic *L. biflexa* serovar Patoc [14]. Similarly, the *rrs* and *lipL32* genes are among the most utilized targets for detecting leptospiral DNA in clinical specimens; however, *rrs*‐based assays amplify both pathogenic and intermediate *Leptospira*, while *lipL32*, though specific to the pathogenic clade, demonstrates lower sensitivity compared with *rrs* [10, 15, 16]. Despite this limitation, *lipL32* remains the predominant target in most commercial diagnostic kits for pathogenic *Leptospira* detection.

To address the limitations of existing diagnostic targets, the present study employed a modified comparative genomics approach to identify a novel sequence conserved exclusively among pathogenic *Leptospira*. Bioinformatic analysis revealed an oxidoreductase gene segment and the terminal region of a hypothetical protein, collectively designated as *orpH*, as a unique marker specific to pathogenic *Leptospira*.

To validate its diagnostic utility, the *orpH* target was evaluated in parallel with the commonly used *lipL32* gene by conventional PCR using DNA from a panel of *Leptospira* and non‐*Leptospira* bacterial strains. Both *PCR‐orpH* and *PCR‐lipL32* demonstrated 100% analytical specificity for 26 *Leptospira* strains, with no amplification observed for saprophytic or heterologous bacterial species. However, *PCR‐orpH* exhibited a superior limit of detection (1 pg/μL) compared with *PCR‐lipL32* (10 pg/μL), indicating a tenfold higher analytical sensitivity. Sequencing of *orpH* amplicons confirmed complete identity with pathogenic *Leptospira* sequences, further verifying target specificity.

Following this confirmation of enhanced analytical performance, the *orpH* target was selected for development of a colorimetric LAMP assay and subsequent evaluation in clinical samples to assess its diagnostic applicability.

In this study, a colorimetric LAMP assay was developed for the specific detection of the *orpH* target (LAMP‐*orpH*). Reaction parameters were systematically optimized, establishing 65°C and 90 min as the optimal incubation temperature and reaction time, respectively. A distinct color change from pink to yellow, visible to the naked eye, indicated successful amplification after 90 min. An intermediate orange coloration was observed at 60 min and subsequently confirmed as a positive result by gel electrophoresis; therefore, a transition from orange to yellow was interpreted as a positive reaction (Figure 2). Although previous reports have described shorter LAMP reaction times of 40–60 min [14, 32], the timeframe was extended to 90 min. The reaction time was extended because the assay employed four primers rather than the conventional six, as additional primers may increase the risk of nonspecific amplification and false‐positive reactions, thereby necessitating a longer incubation period.

A total of 76 human serum specimens from patients with suspected leptospirosis were analyzed to comprehensively evaluate the diagnostic performance of the LAMP‐*orpH*, *PCR‐orpH*, and MAT assays. Their results were compared with a commercial real‐time PCR targeting the *lipL32* gene, an established molecular marker for *Leptospira* spp. The sensitivity and specificity of diagnostic assays are fundamental indicators of their clinical utility.

The LAMP‐*orpH* assay demonstrated the highest sensitivity (87.23%) compared with *PCR‐orpH* (23.40%) and MAT (63.83%), underscoring its effectiveness for early detection of leptospirosis. High sensitivity is critical during the leptospiremic phase, when bacterial loads are low, and antibody responses have not yet developed. In contrast, although PCR‐orpH and MAT exhibited high specificity (98.28%), their lower sensitivity reduced diagnostic yield. The diminished sensitivity of PCR likely reflects the influence of inhibitors present in clinical specimens, which can interfere with amplification and contribute to false‐negative results.

These findings are consistent with a comprehensive meta‐analysis reporting that conventional PCR achieved 54% sensitivity and 88% specificity, whereas LAMP demonstrated superior diagnostic performance with 80% sensitivity and 91% specificity [18], The superior sensitivity observed in our study aligns with analytical results from bacterial strains, where the LAMP‐orpH assay achieved a detection limit of 100 fg/μL tenfold lower than that of PCR‐orpH.

Although MAT remains the gold‐standard serological method for *Leptospira* detection during the immune phase, its reliance on antibody production limits its effectiveness in the early stage of infection, when antibodies are not yet detectable. In this study, MAT demonstrated a sensitivity of 63.83% during the acute phase. In contrast, Danish et al. reported that by the seventh day of illness—when antibodies are typically produced—MAT sensitivity increased to over 90% [37]. This finding underscores the fundamental limitation of serological assays and emphasizes the advantages of molecular diagnostic techniques, such as LAMP, which enable the direct detection of *Leptospira* DNA independent of the host's immune response.

Positive predictive value (PPV) and negative predictive value (NPV) are critical indicators of a diagnostic assay's clinical reliability. The LAMP‐*orpH* assay demonstrated an excellent PPV of 93.18%, signifying that a positive result is highly indicative of true infection—an essential attribute for guiding timely therapeutic intervention. Its high NPV of 90.16% further supports its reliability in correctly identifying uninfected individuals and minimizing false‐negative outcomes. The overall diagnostic accuracy of the LAMP‐*orpH* assay reached 91.43%, substantially outperforming both *PCR‐orpH* (64.76%) and MAT (82.86%), thereby reflecting its superior balance between sensitivity and specificity.

Comparative analysis revealed that *PCR‐orpH* exhibited a lower NPV (61.29%) and poor overall accuracy, consistent with its higher false‐negative rate. Although MAT achieved a high PPV (96.77%), its diagnostic accuracy remained moderate due to reduced sensitivity during the acute phase of infection. Agreement analysis using Cohen's kappa coefficient (*κ*) further underscored these differences: the LAMP‐*orpH* assay demonstrated strong concordance with the reference qPCR (*κ* = 0.826), MAT showed good agreement (*κ* = 0.642), whereas *PCR‐orpH* displayed poor concordance (*κ* = 0.233). Collectively, these results affirm the diagnostic robustness of the LAMP‐*orpH* assay, which—like qPCR—detects *Leptospira* DNA directly and with high analytical precision, offering a rapid, accurate, and field‐deployable alternative for early leptospirosis diagnosis.

In addition to LAMP, other emerging point‐of‐care diagnostic methods have been explored for *Leptospira* detection. Recombinase Polymerase Amplification (RPA) enables rapid amplification at low temperatures with excellent portability, but its widespread application is limited by complex primer design, higher costs, and a greater risk of nonspecific amplification [38, 39, 40, 41, 42]. RPA–CRISPR/Cas platforms build upon RPA by adding CRISPR‐based detection, offering enhanced specificity and multiplexing potential, yet they remain constrained by multi‐step workflows, reagent stability issues, and only moderate sensitivity in clinical contexts, as demonstrated by the *lipL32*‐based assay (85.2% sensitivity, 100% specificity; LOD≈2.1 × 10^3^ cells/mL) [43]. Aptamer‐based assays, such as gold nanoparticle (AuNP) colorimetric systems, provide rapid, low‐cost, and highly specific detection of pathogenic *Leptospira* in water samples, making them attractive for outbreak surveillance but their diagnostic adoption is still hindered by the need for large‐scale validation and regulatory approval [44]. Taken together, while RPA and aptamer‐based methods highlight innovative directions for leptospirosis diagnostics, the single‐step colorimetric LAMP‐orpH assay described here offers a more balanced combination of sensitivity, specificity, and field applicability, underscoring its practical suitability for resource‐limited settings.

## 5 Limitations

There were some limitations to our study that must be acknowledged. In the analytical phase, the evaluation was limited by the lack of multiple serovars from the P2 (intermediate) and S1 (saprophytic) subclades, which prevented a comprehensive assessment of potential cross‐reactivity. In the clinical phase, anonymized samples were used, and therefore information regarding the day of symptom onset was unavailable, which may have influenced the interpretation of diagnostic sensitivity across disease stages. Moreover, paired (convalescent‐phase) serum samples, which are typically required for serological confirmation by MAT, were not available. The limited number of clinical samples also constrained the statistical power of some analyses. Finally, whole blood and urine specimens were not included in this study; such specimens could have provided valuable complementary data to further validate the diagnostic performance of the novel *orpH* target and enhance understanding of its detection dynamics in different biological matrices.

In conclusion, the modified comparative genomic analysis successfully identified the *orpH* sequence as a novel diagnostic target for both PCR and LAMP assays, providing an additional reliable marker for the detection of pathogenic *leptospira* the LAMP‐orpHassay demonstrated high sensitivity, specificity, and diagnostic accuracy with strong agreement between molecular results and clinical diagnoses. These findings indicate that the LAMP‐orpH assay represents a practical and reliable alternative to PCR‐based methods, offering rapid detection without the requirement for a costly PCR machine. This advantage makes it particularly suitable for use in resource‐limited and endemic settings where leptospirosis is prevalent.

This study constitutes the first successful development of a LAMP assay targeting the *orpH* gene, which exhibited superior diagnostic performance compared with the widely used *lipL32* target. Further studies involving larger sample sizes and diverse clinical settings are warranted to validate these findings and assess the assay's broader applicability. Additionally, incorporating the *orpH* sequence into other molecular diagnostic platforms, such as real‐time PCR or microarray assays, may enhance diagnostic accuracy and support improved surveillance of leptospirosis at both clinical and public health levels.

## Author Contributions

Conceptualization: Peyman Ghoraishizadeh and Kulachart Jangpatarapongsa. Methodology: Peyman Ghoraishizadeh and Kulachart Jangpatarapongsa. Investigation: Peyman Ghoraishizadeh, Benjie Clemente, Kween Saimuang, Sakaorat Lerdjuthaporn, Sukhonta limsampan, Patcharapan Suwannin, and Mongkol Techakasikornpanich. Sample collection and preparation: Benjie Clemente, Galayanee Duangchawee, Edilberto Manahan, and Kulachart Jangpatarapongsa. Writing – original draft preparation: Peyman Ghoraishizadeh and Kulachart Jangpatarapongsa. Writing – review and editing: Kulachart Jangpatarapongsa. Supervision: Kulachart Jangpatarapongsa.

## Funding

This work was supported by the Research Assistant scholarship Faculty of Medical Technology, Mahidol University, the Excellent Research Scholarship, Mahidol University, and the Mahidol University Research Strategic Fund.

## Ethics Statement

Ethical approval for all procedures performed in this study involving human participants was in accordance with the ethical standards of the institutional and/or national research committee and with the 1964 Helsinki Declaration and its later amendments or comparable ethical standards. The Institutional Review Board—REC Philippines (reference number: 2024‐027). January 31, 2024.

## Conflicts of Interest

The authors declare no conflicts of interest.

## Supporting information


**Figure S1:** Scheme for modified comparative genomic analysis for *Leptospira*.
**Figure S2:** New target sequence's graphic including *oxidoreductase* and a hypothetical protein.
**Figure S3:** Gel electrophoresis analysis of PCR amplification for evaluating assay specificity and sensitivity.
(A, C) PCR‐*orpH*; (B, D) PCR‐*lipL32*.
**Figure S4:** Analytical sensitivity and limit of detection (LOD) of the LAMP‐*orpH* assay.
**Figure S5:** Analytical specificity of the colorimetric LAMP‐orpH assay.

## Data Availability

The data that support the findings of this study are available from the corresponding author upon reasonable request.
