# Supplementary material for: Novel Target for Pathogenic Leptospira Detection via Colorimetric Loop‐Mediated Isothermal Amplification (LAMP): Development, Validation, and Comparative Analysis
Source: J Clin Lab Anal. 2026 Oct 14:e70277. Online ahead of print. doi: 10.1002/jcla.70277 (PMC13669682; doi:10.1002/jcla.70277)
Supplement: Supplementary file 1 [file JCLA-9999-e70277-s001.docx]

**Figure S1:** **Scheme for modified comparative genomic analysis for *Leptospira*.**

**Figure S2:** **New target sequence’s graphic including *oxidoreductase* and a hypothetical protein**.

**Figure S3:** **Gel electrophoresis analysis of PCR amplification for evaluating assay specificity and sensitivity.
(A, C) PCR-*orpH*; (B, D) PCR-*lipL32*.**

**(A, B):** Panels A and B depict PCR specificity. Lanes: M, 100 bp DNA marker; Cr+, positive control; Cr–, no-template control; lanes 1–9 correspond to pathogenic Leptospira serovars as follows—lane 1: Canicola, lane 2: Panama, lane 3: Djasmen, lane 4: Bratislava, lane 5: Shermani, lane 6: Ranaram, lane 7: Copenhageni, lane 8: Autumnalis, and lane 9: Pomona

**(C, D):** Analytical sensitivity of the PCR assay using Leptospira interrogans serovar Canicola DNA.
Lanes M: 100 bp DNA marker; Cr–: no-template control. Lanes 1–8 represent tenfold serial dilutions of Canicola genomic DNA (100 ng, 10 ng, 1 ng, 100 pg, 10 pg, 1 pg, 100 fg, and 10 fg per reaction, respectively).

**Figure S4. Analytical sensitivity and limit of detection (LOD) of the LAMP-*orpH* assay.
(A, C)** Analytical sensitivity of the LAMP-*orpH* assay using *Leptospira interrogans* serovar Canicola DNA, evaluated by gel electrophoresis (A) and colorimetric detection (C). Lanes: M, 100 bp DNA marker; 1, no-template control (NC); 2–10, tenfold serial dilutions of Canicola DNA corresponding to 100 ng, 10 ng, 1 ng, 100 pg, 10 pg, 1 pg, 100 fg, 10 fg, and 1 fg per reaction, respectively**.
(B, D)** Limit of detection (LOD) of the LAMP-*orpH* assay using spiked serum samples containing tenfold serial dilutions of *Leptospira interrogans* serovar Canicola cells (10⁶ to 1 cell/mL), assessed by gel electrophoresis (B) and colorimetric detection (D). Lanes: M, 100 bp DNA marker; 1–7, serial dilutions (10⁶–1 cell/mL); 8, no-template control (NC); 9, positive control (PC).

**Figure S5:** **Analytical specificity of the colorimetric LAMP-orpH assay.**

Lanes: 1, no-template control (NC); 2–16, pathogenic Leptospira serovars—Canicola, Panama, Djasmen, Bratislava, Shermani, Ranaram, Copenhageni, Autumnalis, Pomona, Ballum, Java, Grippotyphosa, Hardjo, Cynopteri, and Australis, respectively; 17, Leptospira biflexa (saprophytic strain); 18, Leptospira fainei (intermediate strain); 19, Pseudomonas aeruginosa; 20, Salmonella Typhi; 21, Treponema pallidum.
